# Microglial Lectins in Health and Neurological Diseases

**DOI:** 10.3389/fnmol.2018.00158

**Published:** 2018-05-14

**Authors:** Jian Jing Siew, Yijuang Chern

**Affiliations:** ^1^Taiwan International Graduate Program in Molecular Medicine, National Yang-Ming University and Academia Sinica, Taipei, Taiwan; ^2^Institute of Biomedical Sciences, Academia Sinica, Taipei, Taiwan

**Keywords:** microglia, galectin, Siglec, selectin, mannose-binding lectin, neurological disease, neurodegeneration

## Abstract

Microglia are the innate sentinels of the central nervous system (CNS) and are responsible for the homeostasis and immune defense of the CNS. Under the influence of the local environment and cell-cell interaction, microglia exhibit a multidimensional and context-dependent phenotypes that can be cytotoxic and neuroprotective. Recent studies suggest that microglia express multitudinous types of lectins, including galectins, Siglecs, mannose-binding lectins (MBLs) and other glycan binding proteins. Because most studies that examine lectins focus on the peripheral system, the functions of lectins have not been critically investigated in the CNS. In addition, the types of brain cells that contribute to the altered levels of lectins present in diseases are often unclear. In this review, we will discuss how galectins, Siglecs, selectins and MBLs contribute to the dynamic functions of microglia. The interacting ligands of these lectins are complex glycoconjugates, which consist of glycoproteins and glycolipids that are expressed on microglia or surrounding cells. The current understanding of the heterogeneity and functions of glycans in the brain is limited. Galectins are a group of pleotropic proteins that recognize both β-galactoside-containing glycans and non- β-galactoside-containing proteins. The function and regulation of galectins have been implicated in immunomodulation, neuroinflammation, apoptosis, phagocytosis and oxidative bursts. Most Siglecs are expressed at a low level on the plasma membrane and bind to sialic acid residues for immunosurveillance and cell-cell communication. Siglecs are classified based on their inhibitory and activatory downstream signaling properties. Inhibitory Siglecs negatively regulate microglia activation upon recognizing the intact sialic acid patterns and vice versa. MBLs are expressed upon infection in cytoplasm and can be secreted in order to recognize molecules containing terminal mannose as an innate immune defense machinery. Most importantly, multiple studies have reported dysregulation of lectins in neurological disorders. Here, we reviewed recent studies on microglial lectins and their functions in CNS health and disease, and suggest that these lectin families are novel, potent therapeutic targets for neurological diseases.

## Introduction

The central nervous system (CNS) consists of neurons and glial cells such as microglia, astrocytes and oligodendrocytes. Microglia are the CNS counterpart of macrophages and differ from other types of glial cells in terms of their origin, morphology and functions (Kettenmann et al., 2011). They are widely distributed throughout the brain and spinal cord, and constitute 5%–10% of total brain cells depending on the region (Lawson et al., 1990).

Microglia play multiple roles in healthy and diseased brains. During development, microglia regulate the number of neuronal cells by triggering programmed cell death and actively phagocytose the apoptotic neurons (de la Rosa and de Pablo, 2000; Bessis et al., 2007). In addition, microglia are capable of promoting neurogenesis by secreting trophic factors (such as nerve growth factor (NGF) and insulin-like growth factor 1 (IGF-1; Frielingsdorf et al., 2007; Thored et al., 2009). In addition to regulating the number of neurons, microglia also strengthen and prune excessive synapses to ensure that neuronal circuits are connected correctly (Paolicelli et al., 2011). Hence, microglia have been implicated in synaptic plasticity.

As the immune sentinels of the CNS, microglia are responsible for detecting and swiftly responding to infection, injury, molecules released by damaged cells and misfolded proteins (Davalos et al., 2005; Nimmerjahn et al., 2005). In this context, microglia are believed to play exacerbating roles in the inflammatory response of neurological diseases, including multiple sclerosis (MS; Politis et al., 2012), ischemic stroke (Patel et al., 2013), Alzheimer’s disease (AD; Fuhrmann et al., 2010), Parkinson’s disease (PD; Subramaniam and Federoff, 2017), amyotrophic lateral sclerosis (ALS; Brites and Vaz, 2014), Huntington’s disease (HD; Hsiao et al., 2014) and virus infection in the brain (Cenker et al., 2017). Hence, elucidating the machinery that regulates the multidimensional phenotypes of microglia is critical for understanding neurological diseases and developing effective therapeutic interventions.

Microglia express various types of lectins that functionally regulate their dynamic phenotypes. These lectins include galectins, Siglecs, C-type lectins (such as mannose-binding lectins, MBLs) and other glycan binding proteins. Some of these lectins are expressed constitutively, while others are expressed upon stimulation. For instance, microglia generally do not express galectin-3, but its expression can be up-regulated by inflammatory mediators (Burguillos et al., 2015). Galectins have multiple cellular localizations and different functions. For instance, galectin-1, -3 and -9 have been reported to exist in the nucleus, cytoplasm, plasma membrane and extracellular matrix. The cell types in the CNS that express galectins and their cellular localization and functions are summarized in Table 1. Contrary to galectins, all CNS Siglecs (except Siglec-4) are localized on the plasma membrane of microglia. Details about the CNS Siglecs are summarized in Table 2. Similar to Siglecs, selectins are localized on the plasma membrane of endothelial cells. In contrast, MBL is found in the cytoplasm of all brain cells in disease conditions and can be secreted into the extracellular matrix. Details on these C-type lectins are summarized in Table 3. The cell surface contains a complex layer of glycosylated molecules, such as glycoproteins and glycolipids that are recognized by these lectins and store important information for cell-cell communication and response (Brandley and Schnaar, 1986; Groves, 2013; Letschert et al., 2014). Classification of lectins is based on the homology of amino acid sequences and specificity of glycan structures. Their functions have been reviewed in detail elsewhere (Varki et al., 2009; Fujimoto et al., 2014). In this review, we focus on the role of lectins and how they affect the functions of microglia in the CNS. Tables 1–3 summarizes the major lectins that have been reported in the CNS so far.

**Table 1:** Summary of galectins in central nervous system (CNS).

Type	Ligands	Cell type/Subcellular location		Functions	References
Galectin-1	β-Galactosides	Microglia Astrocytes Neurons	Nucleus Cytoplasm Plasma membrane Extracellular	Suppression of microglial activation Regulation of neurogenesis Regulation of oligodendrocyte proliferation	Sakaguchi et al. (2006, 2007) and Starossom et al. (2012)
Galectin-3	β-Galactosides	Microglia	Nucleus Cytoplasm Plasma membrane Extracellular	Microglial activation Promotion of neuroinflammation Suppression of neuroinflammation Regulation of oligodendrocyte differentiation	Jiang et al. (2009), Lerman et al. (2012) and James et al. (2016)
Galectin-4	β-Galactosides	Neurons	Plasma membrane	Axonal growth Axonal transport Suppression of axonal myelination	Cao and Guo (2016)
Galectin-8	β-Galactosides	Microglia Others	High levels in hippocampus and choroid plexus Other brain regions CSF	Immunosuppression	Stancic et al. (2011), John and Mishra (2016) and Pardo et al. (2017)
Galectin-9	β-Galactosides	Microglia Astrocytes	Nucleus Cytoplasm Plasma membrane Extracellular	Modulation of neuroinflammation	Stancic et al. (2011), Lerman et al. (2012) and Steelman et al. (2013)

**Table 2:** Summary of Siglecs in CNS.

Type	Ligands	Cell type/ Subcellular location		Functions	References
Siglec-1 (sialoadhesin, CD169)	α2–3, α2–6 sialic acid residues	Microglia	• Plasma membrane	• Cell-cell interaction	Macauley et al. (2014) and Groh et al. (2016)
Siglec-3 (CD33)	α2–6, α2–3 sialic acid residues	Microglia	• Plasma membrane	• Modulation of microglial phagocytosis activity	Griciuc et al. (2013) and Macauley et al. (2014)
Siglec-4 (MAG)	α2–3, α2–6 sialic acid residues	On myelin sheath, produced by oligodendrocytes	• Inner-most, myelin periaxonal membrane	• Myelin-axon interaction, maintaining long-term axon stability	Sun et al. (2004), Huang et al. (2012) and Macauley et al. (2014)
Siglec-E	α2–3, α2–6, α2–8 sialic acid residues	Microglia	• Plasma membrane	• Inhibition of inflammation, ROS production and phagocytosis	Claude et al. (2013) and Macauley et al. (2014)
Siglec-F	α2–3 sialic acid residues	Microglia	• Plasma membrane	• Inhibition of phagocytosis	Wielgat and Braszko (2012) and Macauley et al. (2014)
Siglec-H	Abnormal sialic acid residues in glioma	Microglia	• Plasma membrane	• Promotion of phagocytosis	Kopatz et al. (2013) and Macauley et al. (2014)
Siglec-11	α2–8 sialic acid residues	Microglia	• Plasma membrane	• Inhibition of inflammation and phagocytosis	Wang and Neumann (2010), Wang X. et al. (2012) and Macauley et al. (2014)
Siglec-16	α2–8 sialic acid residues	Microglia	• Plasma membrane	• No functional data	Wang X. et al. (2012) and Macauley et al. (2014)

**Table 3:** Summary of C-type lectins in CNS.

Type	Ligands	Cell type/Subcellular location		Functions	References
E-Selectin	Sialyl-Lewis X terminal	Endothelial cells	Plasma membrane	Facilitate the adhesion of neutrophils	Jin et al. (2010)
P-Selectin	Sialyl-Lewis X terminal	Endothelial cells	Plasma membrane	Facilitate the adhesion of neutrophils	Atkinson et al. (2006) and Jin et al. (2010)
Mannose-binding lectin	Terminal mannose, fucose, GlcNAc	Microglia Astrocytes Oligodendrocytes Neuron	Cytoplasm Extracellular	Innate immune defense	Singh et al. (2011)

## Galectins

Galectins represent a family of lectins characterized by conserved carbohydrate-recognition domains (CRDs) and bind to β-galactosides with different specificities and affinities (Liu and Rabinovich, 2005). Through the conserved CRDs, 15 galectins have been identified; they can be categorized into three groups: (1) proto, or one CRD; (2) tandem repeats, or two distinct CRDs in tandem; and (3) chimera, consisting of CRD with unusual tandem repeat of glycine- and proline-rich short stretches (Kasai and Hirabayashi, 1996). The prototype galectins include galectins-1, -2, -5, -7, -10, -11, -13, -14 and -15, while the tandem repeats type consists of galectins -4, -6, -8, -9 and -12; and galectin-3 is the only galectin classified as chimera type. Among these galectins, only galectins -1, -2, -3, -4, -7, -8, -9, -10, -12 and -13 are expressed in humans (Chang et al., 2017). Furthermore, many of these galectins have differentially spliced forms that may be expressed in different tissues. For instance, galectins-8 and -9 have seven and three isoforms, respectively (Zhang et al., 2009; Troncoso et al., 2014). In particular, the galectin-9M and 9S isoforms positively promote the expression of E-selectin, while the galectin-9L isoform suppresses the level of E-selectin (Zhang et al., 2009). These isoforms are critical in regulating important cellular activity. The mechanisms involved in coordinating the expression of these isoforms and the functions they serve are still in need of extensive exploration. To date, no specific surface receptor for galectins has been reported. Nonetheless, galectins can bind to and interact with various glycoproteins and cell membrane glycoconjugates via their carbohydrate moieties and trigger a cascade of transmembrane signaling events (Liu and Rabinovich, 2005; Wan and Liu, 2016). In addition, galectins have non-carbohydrate binding partners and function intracellularly to regulate gene transcription, mRNA splicing, cell growth, apoptosis and immune responses (Liu et al., 2002; Wang J. L. et al., 2012). For instance, intracellular galectin-1 and galectin-3 interact with Gemin4 to facilitate the assembly of spliceosomes (Park et al., 2001). The functions of galectins in the peripheral system have been extensively studied. To date, only galectins-1, -3, -4, -8 and -9 have been found in the CNS. Table 4 summarizes the role of galectins in neurological disorders.

**Table 4:** Summary of the role of galectins in neurological disease.

Neurological conditions	Type of galectin	Disease-associated features	References
Multiple sclerosis	Galectin-1	Astrocytes produce galectin-1 to deactivate microglia through the p38, CREB and NF-κB pathways. The function of galectin-1 produced by MS microglia is unknown.	Starossom et al. (2012)
	Galectin-3	Modulation of immune cell infiltration into the CNS Up-regulation of inflammatory cytokines and down-regulation of anti-inflammatory cytokines in dendritic and T cells. Inhibition of neural cell proliferation	Jiang et al. (2009) and James et al. (2016)
	Galectin-8	Up-regulated in microglia. Immunosuppression in the periphery, unknown effects in the brain.	Stancic et al. (2011) and Pardo et al. (2017)
	Galectin-9	Up-regulated in CSF of secondary progressive MS patients and microglia, its function is not clear. Located in the cytosol of microglia in inactive lesions. Located in the nuclei and cytosol of microglia in active lesions.	Stancic et al. (2011) and Burman and Svenningsson (2016)
Stroke and ischemia	Galectin-3	Up-regulated in the microglia, promotes proliferation. Promotes the production of IGF-1. Suppression of IL6 inflammatory cytokine. Acts as the ligand of TLR4 to promote inflammatory responses.	Lalancette-Hébert et al. (2007, 2012) and Burguillos et al. (2015)
Traumatic brain injury	Galectin-3	Up-regulated in the corpus callosum and is associated with the production of NGF. Up-regulated in the cortex and hippocampus and promotes inflammation.	Venkatesan et al. (2010) and Yip et al. (2017)
Amyotrophic Lateral Sclerosis (ALS)	Galectin-1	Up-regulated in the spinal cord at the end stage.	Lerman et al. (2012)
	Galectin-3	Up-regulated in the microglia and suppresses inflammation and oxidative damage in the spinal cord.	Lerman et al. (2012)
	Galectin-9	Up-regulated in the spinal cord at the symptomatic stage	Lerman et al. (2012)
Parkinson’s disease	Galectin-3	Up-regulated in the microglia to promote phagocytosis of α-synuclein and secretion of inflammatory cytokines.	Boza-Serrano et al. (2014)
Prion disease	Galectin-3	Up-regulated in the medulla and pons. Up-regulation associated with impairment of lysosomal function and autophagy.	Riemer et al. (2004) and Mok et al. (2007)

### Galectins and Myelination

**Figure 1:**
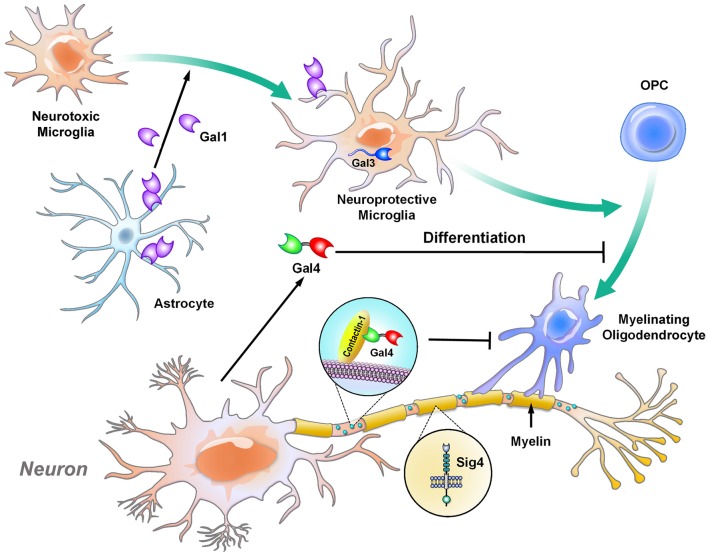
Galectins and Siglec-4 contribute to the glia-mediated regulation of neuronal myelination. Oligodendrocytes are the cells responsible for myelination in the central nervous system (CNS). Under normal conditions, galectin-4 is delivered to the unmyelinated axonal regions and co-localizes with contactin-1 (a marker for nodes of Ranvier). The presence of galectin-4 inhibits oligodendrocytes from myelinating the region (Çolakoğlu et al., 2014). In addition, soluble galectin-4 inhibits the maturation of oligodendrocytes (Stancic et al., 2012). Siglec-4 is produced by oligodendrocytes and is expressed in the myelin sheath (Sun et al., 2004; Huang et al., 2012). Astrocytes and microglia promote the proliferation and differentiation, respectively, of oligodendrocytes. In particular, astrocytes secrete galectin-1 to drive microglia toward neuroprotective phenotype, while these microglia trigger the differentiation of oligodendrocytes in a galectin-3-dependent manner (Hoyos et al., 2014, 2016). Gal, galectin; Sig, Siglec.

During the maturation of neurons, axons are myelinated in a specific manner that leaves certain parts of the segments unmyelinated; these unmyelinated nodes are marked by contactin-1 (Çolakoğlu et al., 2014; Figure 1). Beginning in development, the expression of galectin-4 is different in each cortical layer and brain region. Galectin-4 is absent in the striatum and cortical layer VI, which are heavily myelinated, while its level remains high in cortical layers II to III, which contain neurons with less myelination at the mature stage. In particular, galectin-4 is found to cover these unmyelinated segments, colocalize with contactin-1 and inhibit oligodendrocytes from depositing myelin on these segments (Díez-Revuelta et al., 2017). Additionally, galectin-4 is secreted by neurons and inhibits the maturation of oligodendrocytes (Stancic et al., 2012). In contrast, astrocytes and microglia are involved in regulating and supporting myelination in different manners. In particular, astrocytes promote the proliferation of oligodendrocytes, while microglia induce the differentiation of oligodendrocytes, as demonstrated by culturing oligodendrocytes with conditioned medium from the two cell types (Pang et al., 2013). In addition, galectin-1, which is secreted mainly by astrocytes, shift microglia from an inflammatory to an anti-inflammatory status, which subsequently drives oligodendrocytes toward the myelination process (Starossom et al., 2012; Miron et al., 2013; Sirko et al., 2015). In parallel with these findings, galectin-3 controls the integrity of myelin *in vivo*, as demonstrated by the loosely wrapped myelin structures in galectin-3-depleted mice (Pasquini et al., 2011). Furthermore, in the cuprizone-induced demyelination mouse model, the level of galectin-3 is increased in microglia, which promotes the differentiation of oligodendrocytes for remyelination. The abovementioned machinery is impaired in galectin-3-depleted mice (Hoyos et al., 2014, 2016).

### Galectins in Multiple Sclerosis (MS)

MS is a chronic inflammatory and autoimmune neurological disease marked by damage to the myelin that surrounds the axons in the CNS (Goldenberg, 2012). The pathogenesis of MS can be divided into two processes: (1) the overactivated immune system damages the myelin sheath and axon; and (2) remyelination is impaired (Dendrou et al., 2015). Both microglia and macrophages are chronically activated throughout the progression of MS and contribute to the inflammatory and demyelination processes (Fischer et al., 2012). Importantly, galectins-1, -3, -8 and -9 are reported to be up-regulated in human MS white matter samples. Immunohistochemical staining reveals that galectin-1 is up-regulated in microglia and macrophages, while its quantity is decreased in the astrocytes, at the lesioned area. The reduction of astrocytic galectin-1 may be due to the enhanced release of galectin-1 by astrocytes and may play an important role in MS progression. Conversely, the levels of galectins-8 and -9 are increased in microglia and macrophages located in active MS lesions. Interestingly, galectin-9 alters cellular localization in MS microglia upon activation. In inactive lesion, galectin-9 is located in the cytoplasmic region of microglia, while it is present in both the nuclei and the cytosol of microglia in active lesions (Stancic et al., 2011). The functional relevance of this nuclear localization of galectin-9 remains elusive and requires further investigation.

**Figure 2:**
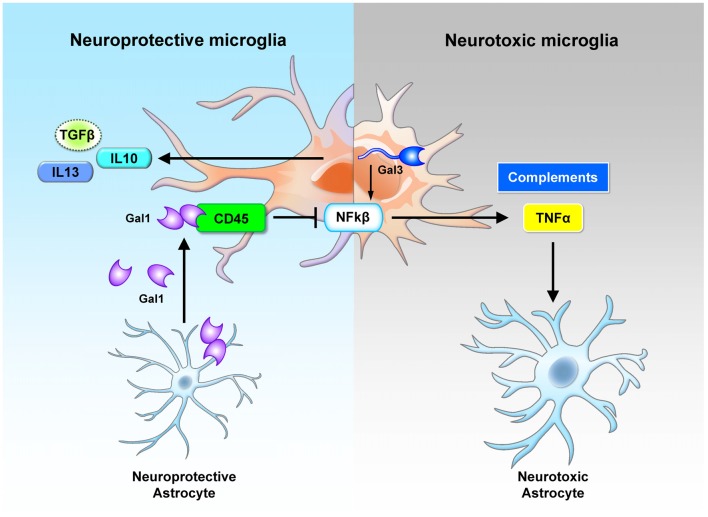
Cross-regulation between astrocytes and microglia by galectin-1 and galectin-3. Neuroprotective astrocytes secrete galectin-1 to inhibit the neurotoxic activation of microglia. The secreted galectin-1 binds with CD45 on the microglia surface, suppresses NF-κB activation and promotes the polarization of neuroprotective microglia (Starossom et al., 2012). Neurotoxic microglia express various types of lectins. In particular, the up-regulated galectin-3 promotes inflammation response through NF-κB activation (Boza-Serrano et al., 2014). These microglial cells secrete inflammatory cytokines (TNFα) and complements that trigger the activation of neurotoxic astrocytes (Liddelow et al., 2017). Gal, galectin; Sig, Siglec.

To date, the most widely used animal model for the study of MS pathogenesis has been the experimental autoimmune encephalomyelitis (EAE) model (Steinman and Zamvil, 2006). In the EAE model, the expression of galectin-1 is up-regulated, with the highest level at the peak of the disease. Further analysis shows that galectin-1 is highly expressed in astrocytes (GFAP+ cells) and moderately expressed in a subset of T cells (CD4+ cells) and microglia/macrophages (CD11b+ cells) during the acute phase. Interestingly, astrocytes, but not other cell types, continue to express galectin-1 during the chronic phase. One of the specific binding targets of the secreted galectin-1 consists of the core 2 O-glycans of CD45 on the cell surface of microglia. Treatment of the pro-inflammatory microglia with galectin-1 inhibits the activation of the p38, CREB and NF-κB pathways (Figure 2). In the absence of galectin-1, MS progresses much more rapidly. More microglia with inflammatory phenotype and much more severe demyelination can be observed in the galectin-1 depleted EAE model (Starossom et al., 2012). These studies suggest a beneficial role of galectin-1 in the EAE model.

The level of galectin-3 is also up-regulated in the phagocytosing microglia in areas of demyelination in the EAE model (Reichert and Rotshenker, 1999; Stancic et al., 2011). Further studies suggested that the onset and severity of EAE were markedly reduced in galectin-3 KO mice (Jiang et al., 2009). In a demyelination experimental model of MS, generated by infection with Theiler’s murine encephalomyelitis virus (TMEV), the level of galectin-3 is also up-regulated in microglia residing in the subventricular zone (the neurogenetic niche that is markedly inflamed in MS and its murine models) and the cerebral cortex. In this model, genetic removal of galectin-3 greatly normalizes the immune response observed in the SVZ, reduces the migration of immune cells into the SVZ, and increases the number of progenitors in the corpus callosum (James et al., 2016). These lines of evidence suggest that galectin-3 may play an essential role in MS.

Galectin-8 can be detected in the cerebrospinal fluid (CSF) of MS patients. Genetic removal of galectin-8 hastens the onset of the disease and worsens the progression. Daily treatment with galectin-8 significantly delays the disease progression of EAE. In particular, galectin-8 inhibits the activity of Th17-positive cells to induce inflammation by influencing cells toward apoptosis (Pardo et al., 2017). Similarly, galectin-9 can also be detected in the CSF of secondary progressive MS patients (Burman and Svenningsson, 2016). Compared to the peripheral nervous system, the roles of galectin-8 and galectin-9 in the CNS (such as their expression profiles and their functions in different brain cells) have not been extensively explored and are worth further investigation.

### Galectins in Stroke and Ischemia

Galectin-3 is up-regulated in microglia after stroke and induces the expression of IGF-1 (Lalancette-Hébert et al., 2007). Importantly, galectin-3 interacts directly with IGF-1 to promote post-ischemic microglial proliferation. These microglia are suggested to serve as the reservoir of neurotrophic factors, such as IGF-1. Genetic depletion of galectin-3 significantly reduces microglial activation and proliferation; these effects are associated with larger ischemic lesion size, decreased IGF-1 levels and neuronal death. Moreover, there is a twofold increase in IL6 in the ischemic brains of galectin-3-depleted mice (Lalancette-Hébert et al., 2012). These studies suggest that the presence of galectin-3 promotes alternative activation of microglia toward an anti-inflammatory status after stroke. Nonetheless, in a separate study, secreted galectin-3 was shown to act as a ligand for the toll-like receptor 4 (TLR4) and promote inflammatory response in a murine neuroinflammatory LPS model and in the brain of stroke patients. Compared to wild-type controls, galectin-3-knockout (KO) mice have elevated neuronal survival in the hippocampus after ischemia, suggesting that galectin-3 prolongs microglia at the pro-inflammatory status (Burguillos et al., 2015). Consistent with a detrimental role, galectin-3 is found to be up-regulated in the CA1 and CA2 hippocampal regions following transient ischemia. The expression of galectin-3 in these regions is associated with neuronal death and can be prevented by intra-ischemic hypothermia treatment (Satoh et al., 2011; Hisamatsu et al., 2016). Moreover, in the permanent middle cerebral artery occlusion (pMCAO) model, the lack of galectin-3 rescues significant neuronal loss. Treatment with sera collected from pMCAO-treated WT mice also induces increased death of enteric neurons compared with sera collected from pMCAO-treated galectin-3 KO mice. The death of these neurons can be prevented by inhibition of TAK1 and AMPK (transforming growth factor and beta-activated kinase 1 and AMP activated kinase, respectively; Cheng et al., 2016). Collectively, the inflammatory roles of galectin-3 are diverse and may depend on the following factors: the type of ischemic insult, the stage of disease/trauma and the subcellular localization.

### Galectins in Traumatic Brain Injury (TBI)

Traumatic Brain Injury (TBI) is clinically categorized into three groups, namely, mild, moderate and severe. Its effects can be further divided into primary and secondary injuries. Primary injuries (such as direct damage to the brain parenchyma that may shear axons) are usually irreversible. On the other hand, secondary injuries are the detrimental effects resulting from the primary injury (such as inflammation and ischemia; Blennow et al., 2016). In response to TBI, in order to restore the normal brain environment, microglia rapidly migrate to the site of injury to remove damaged and dead cells. In a mouse closed-skull model of TBI, the level of galectin-3 increased in the corpus callosum for about 1 month following injury, with the peak at 24 h after injury. The galectin-3- positive microglia engulf damaged axons and produce NGF that facilitates the repair of damaged neurons (Venkatesan et al., 2010). In another study using the controlled cortical impact (CCI) model of head injury, galectin-3 is also up-regulated in the microglia but binds with TLR4 to promote inflammation in the cortex and hippocampus. Treatment with a galectin-3-neutralizing antibody reduces the expression of pro-inflammatory genes *IL1β*, *IL6* and *NOS2* while promoting the expression of *Ym1*, *Arg1* and *TGFβ*. Consistent with a damaging role of galectin-3, galectin-3-deficient mice show less neuronal death and microglial activation in a CCI model (Yip et al., 2017). These data suggest that the role of microglial galectin-3 in TBI can be complex depending on the severity and type of trauma.

### Galectins in Amyotrophic Lateral Sclerosis (ALS)

ALS is a rare, progressive and fatal neurodegenerative disease. Multiple genes have been identified as being associated with familial ALS, but those genes explain only approximately 10% of cases. Nearly 90% of ALS cases are sporadic (Abhinav et al., 2007). Up-regulation of plasma galectin-3 is detected in ALS patients with limb onset or disease duration longer than 12 months. The level of plasma galectin-3 is correlated with the duration of disease. Female patients exhibit higher increase of galectin-3 in the plasma than male patients, although the reason is still unknown (Yan et al., 2016). A study using the B6SJL SOD1(G93A) transgenic mouse model of familial ALS (Smittkamp et al., 2008) revealed that microglia undergo a transition stage from the protective phenotype in early-stage ALS to the detrimental phenotype in end-stage ALS. Furthermore, early-stage microglia promote the survival of motor neurons in a co-culture system, while end-stage microglia trigger neuronal death (Liao et al., 2012).

In particular, galectin-1 is increased in the spinal cord of SOD1 mice, while galectins-3 and -9 are increased in SOD1 mice and sporadic ALS patients. The alterations in these galectins during ALS progression are different. Specifically, the amount of galectin-3 can be detected in microglia of spinal cord at the presymptomatic stage, and its level continues to increase until the end stage. Conversely, the expression of galectin-9 is increased at the symptomatic stage, while the levels of galectin-1 is up-regulated only at the end stage (Lerman et al., 2012). The types of cells that contribute to the higher levels of galectin-1 and -9 in the spinal cord are currently unknown.

Nonetheless, it is of great interest to observe that treatment of SOD1 mice with recombinant galectin-1 significantly enhances the survival of motor neurons, delays the onset of disease, improves motor performance, and prolongs the lifespan of ALS mice. Because galectin-1 treatment has no specific effect on isolated primary neurons, the therapeutic effect of galectin-1 may be mediated by non-neuronal cells (Chang-Hong et al., 2005). In particular, microglia and macrophages are among the target cells of galectin-1. For example, treatment with galectin-1 induces macrophages to produce an axonal regeneration-promoting factor that is critical to the survival of degenerating motor neurons (Horie et al., 2004). Overall, although the underlying mechanism remain to be clarified, galectin-1 is a potential therapeutic target for ALS.

Another interesting drug target for ALS is galectin-3. Genetic depletion of galectin-3 in SOD1 mice accelerates disease progression (i.e., a shorter life span and faster motor function impairment) with a significant increase in microglial activation, along with higher inflammatory response and oxidative damage (Lerman et al., 2012). In the CNS of healthy rats, the basal expression of galectin-3 is relatively higher in the spinal cord than in the cortex. Flow cytometry analysis detects at least two major populations of microglia (namely, galectin-3- positive and negative) in the spinal cord of SOD1(G93A) rats after disease onset. Compared to microglia in wild-type rats, microglia in the spinal cord of SOD1(G93A) rats express significantly higher levels of galectin-3 from early to late stage of the disease. The levels of both neurotoxic and neuroprotective molecules (i.e., TNFα, IL6, arginase-1, IL10 and BDNF) in the late stage of the disease are lower, compared with in the early stage. Intriguingly, these alterations only appeared in the spinal cord but not in the cortex, suggesting that the function of galectin-3 and the features of microglia are different in the spinal cord and cortex (Nikodemova et al., 2014). Collectively, these findings suggest that microglial galectin-3 plays a protective role in ALS, which appears different from most of the neurodegenerative diseases where the role of galectin-3 has been investigated so far; further investigation is required.

### Galectin-3 in Parkinson’s Disease (PD)

The pathology of PD is characterized by the degeneration of dopaminergic neurons in the substantia nigra and widespread Lewy bodies that contain α-synuclein (α-Syn) aggregates. Accumulated evidence suggest that neurons can release α-Syn to neighboring astrocytes and microglia (Kim et al., 2013). Microglia would take up α-Syn either by phagocytosis or endocytosis. Treatment of a BV2 microglial cell line with different forms of α-Syn (monomer and aggregates) promotes microglial activation as assessed by the levels of iNOS and pro-inflammatory cytokines (TNFα, IL2 and IL12). Importantly, knockdown of galectin-3 or treatment with a galectin-3 inhibitor abolishes the effect of α-Syn on the release of iNOS and pro-inflammatory cytokines. Inhibition of galectin-3 reduces the phagocytic capability of microglia to take up exogenous α-Syn, while treatment with recombinant galectin-3 promotes phagocytosis. Injection of oligomeric forms of α-Syn into the olfactory bulb also significantly enhances the expression of galectin-3 and the phagocytotic activity of microglia. These findings suggest that exposure of microglia to α-Syn causes up-regulation of galectin-3 and subsequently enhances phagocytosis (Boza-Serrano et al., 2014).

**Figure 3:**
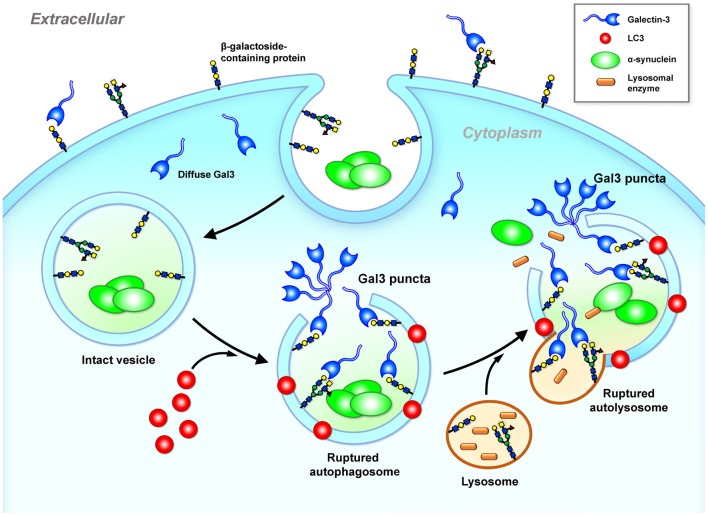
Galectin-3 is a sensor of ruptured intracellular vesicles. Galectin-3 recognizes and binds to β-galactosides. β-galactosides containing proteins are located in the plasma membrane outer leaflet and the luminal side of intact intracellular vesicles (e.g., lysosome, autophagosome and autolysosome). Once these vesicles leak or rupture under stress, galectin-3 gains access and binds to β-galactosides located inside of ruptured vesicles and forms puncta (Freeman et al., 2013; Flavin et al., 2017). Gal3, galectin-3; α-Syn, α-synuclein.

The glycan binding targets of galectin (i.e., β-galactosides) are located in the plasma membrane outer leaflet and the luminal side of the intracellular vesicles. Cytosolic galectins (e.g., galectin-3) therefore have no access to β-galactosides. In a neuronal model (SY5Y cells), treatments with α-Syn trigger the formation of galectin-3 puncta because the endocytosed α-Syn cannot be effectively degraded and triggers cellular stress (e.g., elevated ROS), leading to vesicle rupture (Flavin et al., 2017). Exposure of surface glycans from the ruptured vesicular membranes to the cytoplasm recruits galectin-3, which forms galectin-3 puncta (Freeman et al., 2013; Flavin et al., 2017). Such endosomal and lysosomal ruptures not only expose the internal compartment of the vesicles but also release into the cytoplasm substances (e.g., cathepsin B) that need to be confined inside the vesicle, potentially inducing the mitochondrial dysfunction and inflammation that are the common features of PD. It is likely that cell-to-cell transmission of cytosolic protein aggregates (e.g., α-Syn) may induce cellular stress that causes vesicle rupture and the formation of galectin-3 puncta as illustrated in Figure 3. Similar to galectin-8 (Thurston et al., 2012), galectin-3 may serve as a cellular danger receptor that senses detrimental conditions during neuronal degeneration. Further investigation is required to determine whether the formation of galectin-3 puncta at these ruptured vesicles affects cellular functions.

### Galectin-3 in Prion Diseases

Prion diseases consist of a group of neurodegenerative disorders that are caused by misfolded proteins called prions. Prions are infectious and the disease can occur in a sporadic, genetic or acquired manner. Genetic prion disease is caused by the mutation of prion-related protein gene (*PRPN*; Geschwind, 2015). In the scrapie-infected mouse model of prion disease, gene array analysis of the brain reveals 114 altered mRNA levels, and many of the identified genes were related to immune and stress responses. Among the genes with altered expression, the mRNA of galectin-3 is significantly up-regulated in the medulla and pons (Riemer et al., 2004). In a separate study, the protein level of galectin-3 was also found to be increased in scrapie-infected brains. Because galectin-3 KO mice with scrapie infection survive longer than the scrapie-infected wild-type mice and because the levels of a lysosomal activation marker (LAMP-2) and autophagy-related proteins (i.e., Beclin-1 and Atg5) are significantly decreased in the scrapie-infected galectin-3 KO mice, it is possible that galectin-3 may play a detrimental role by damaging the integrity of lysosomes and suppressing autophagy in prion disease (Mok et al., 2007).

## Siglecs

Siglecs (sialic acid-binding immunoglobulin-like lectins) represent the best-characterized subgroup of I-type lectins, which contain the immunoglobulin domain that recognizes structural diversity of carbohydrates (Crocker et al., 2007). In general, Siglecs are expressed on plasma membrane, containing an amino terminal V-set immunoglobulin domain that binds sialic acid and a number of C2-set immunoglobulin domains. As their full name suggests, Siglecs recognize sialic acid residues (best described as N-acetyl-neuraminic acids, Neu5Ac) that are presented on glycoproteins and glycolipids with distinct yet overlapping specificities. The presence of C-2 α-hydroxyl group at the terminal of sialic acid enables covalent linkage to other hydroxyl groups on the C-3 and C-6 positions to form the α2–3 and α2–6 linkages, respectively. Enzymes that are responsible for generating such linkages are named sialyltransferases. There are multiples types of sialyltransferases that produce a variety of linkages that can be recognized by Siglecs with different specificities.

Currently, up to 16 Siglecs have been identified and can be categorized based on their sequence similarity, conservation among murine and human and the regulatory properties (i.e., inhibitory or activatory; for review, see Pillai et al., 2012; Macauley et al., 2014). In brief, Siglec-1 (sialoadhesin), Siglec-2 (CD22), Siglec-4 (myelin-associated glycoprotein, MAG) and Siglec-15 are conserved across mammals. Conversely, a variety of Siglecs-3 (CD33)- related Siglecs have been found across mammals. The human CD33-related Siglecs include Siglecs-3, -5, -6, -7, -8, -9, -10, -11, -12, -14 and -16, while the murine CD33-related Siglecs consist of Siglecs-3, -E, -F, -G and -H (Macauley et al., 2014). Most Siglecs (excluding Siglec-1 and MAG) contain immunoreceptor tyrosine-based inhibitory motifs (ITIMs) at the cytoplasmic regions to recruit SH2 domain-containing protein tyrosine phosphatase SHP1 and SHP2; these Siglecs are classified as inhibitory Siglecs. On the other hand, Siglecs-14, -15 and -16 contain the immunoreceptor tyrosine-based activation motifs (ITAMs) on the adaptor of DAP12 to recruit spleen tyrosine kinase (SYK); these Siglecs are classified as activatory Siglecs.

Siglecs are expressed specifically on the cells from hematopoietic lineage, with the exceptions of MAG on oligodendrocytes and Siglec-6 on placental trophoblasts (Macauley et al., 2014). Knowledge of Siglecs is collected mainly from the peripheral system, while Siglecs that are reported to exist in the CNS include sialoadhesin, CD33, MAG, Siglec-E, Siglec-F, Siglec-H, Siglec-11 and Siglec-16. Siglecs are generally absent or scarce under normal physiological condition and are induced to express by various stimuli that activate microglia. These Siglecs, with exception of MAG that is located in oligodendrocytes, are expressed on microglia in the brain. Table 5 summarizes the role of Siglecs in neurological disorders.

**Table 5:** Summary of the role of Siglecs in neurological disease.

Neurological conditions	Type of Siglec	Disease-associated features	References
Traumatic brain injury	Siglec-1	• Up-regulated in the microglia upon exposure to plasma proteins, unclear function.	Perry et al. (1992)
Ceriod lipofuscinoses	Siglec-1	• Up-regulated in the microglia of the optic nerve.	Groh et al. (2016)
		• Serves as an APC and interacts with CD8+ T cells to promote inflammation.
Alzheimer’s disease	CD33	• Up-regulated in the microglia to suppress phagocytosis of Aβ.	Bradshaw et al. (2013) and Griciuc et al. (2013)
Brain tumor	Siglec-H	• Phagocytosis of glioma cells	Kopatz et al. (2013)
Amyotrophic lateral sclerosis	Siglec-H	• Up-regulated over time in the spinal cord of SOD1 mice, uncharacterized function.	Chiu et al. (2013)

### Siglec-1 (Sialoadhesin, CD169) During the Breakdown of the BBB

Siglec-1 does not have the ITIM or ITAM at its cytoplasmic regions but contains a long immunoglobulin domain that allows the binding domain to reach a distal site from the membrane. Therefore, Siglec-1 is thought to be important for cell-cell adhesion and cell-pathogen recognition (Crocker et al., 1994). Siglec-1 is present in peripheral blood mononuclear phagocytes. Nevertheless, its expression in microglia is rather controversial and may be due to the exposure to plasma proteins upon the breakdown of the blood-brain barrier (BBB) during brain trauma (e.g., TBI model, Perry et al., 1992). Similarly, during retinal degeneration, activated microglia may express Siglec-1 after the breakdown of the blood-retinal barrier (Hughes et al., 2003). Whether these Siglec-1-expressing microglia are originated in the brain or blood-derived cells (i.e., macrophages) that migrate into the brain and their functions are not clear at this time.

In ceroid lipofuscinosis (CLN), a disease that is characterized by lysosomal storage dysfunction, neurodegeneration and early death, the expression of Siglec-1 is significantly up-regulated. These Siglec-1-expressing microglia serve as antigen-presenting cells (APCs) to interact with CD8+ T cells and promote the pathology of the disease. In Siglec-1-deficient CLN mice, microglia express significantly lower inflammatory cytokines (e.g., IL1β and TNFα) and higher level of anti-inflammatory cytokines (e.g., TGFβ). In addition, Siglec-1-deficient CLN mice exhibit reduced axonal degeneration and longer lifespan, supporting a harmful role of Siglec-1 (Groh et al., 2016). Hence, antagonizing the function of Siglec-1 appears as a potential therapeutic approach during the breakdown of the BBB from disease or injury.

### Siglec-3 (CD33) in AD

**Figure 4:**
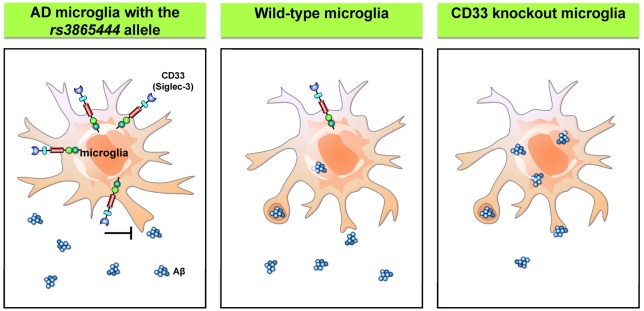
Siglecs regulate microglial phagocytosis. Wild-type microglia express a low level of CD33/Siglec 3, a member of the Siglec family. The risk allele *rs3865444* was originally identified in patients with Alzheimer’s disease (AD) and is associated with higher levels of CD33 that suppress the microglial phagocytosis. Conversely, knockdown of CD33 significantly improves the phagocytosis capability of microglia (Hollingworth et al., 2011; Naj et al., 2011; Griciuc et al., 2013). Aβ, amyloid β.

AD is a chronic neurodegenerative disorder that affects up to 30% of the population over 65 years old (Masters et al., 2015). The pathology of the disease is characterized by intracellular neurofibrillary tangles and failure to clear extracellular amyloid-β (Aβ) peptides from the brain. Most patients with AD have the sporadic form, while a small portion of patients carry genes that cause ineffective clearing of Aβ. Recent genome-wide association studies have identified CD33 as a genetic risk factor of AD (Hollingworth et al., 2011; Naj et al., 2011). The risk allele *rs3865444* is associated with higher expression of CD33 on the cell surface without affecting the number of microglial cells. Moreover, these CD33-immunoreactive microglia exhibit a positive correlation with Aβ aggregates and plaque burden in the brain of AD patients (Bradshaw et al., 2013; Griciuc et al., 2013). Further *in vitro* studies confirmed the presence of CD33 with the risk allele *rs3865444*, which significantly suppresses the phagocytosis capability of microglia. Interestingly, the levels of CD33 with the minor allele of *rs3865444* single nucleotide polymorphism (SNP) are markedly reduced. Similarly, primary microglia derived from CD33 KO mice showed greater uptake of Aβ than the control cells, while the rates of Aβ degradation are unaffected. Moreover, the KO of CD33 in the APP/PS1 mouse model significantly reduces Aβ plaques in the cortex and hippocampus (Griciuc et al., 2013). Therefore, tuning the capability of microglia to take up and degrade Aβ through inhibition of CD33 activity may provide a novel therapeutic intervention for AD. The regulation of microglial phagocytosis by CD33 is summarized in Figure 4.

### Siglec-H in Brain Tumors

Siglec-H is expressed only in murine microglia and plasmacytoid dendritic cells (pDCs), while they are absent in human monocytes (Zhang et al., 2006; Kopatz et al., 2013). In contrast to most Siglecs, Siglec-H is an activatory Siglec with complex functions. Siglec-H suppresses the downstream signaling of TLR9 to produce interferon-α in pDCs (Takagi et al., 2011; Puttur et al., 2013). Although Siglec-H is known to be expressed in microglia, its ligands and functions are only now being discovered, as it does not bind to the typical sialic acid-containing glycoproteins or glycolipids of healthy cells (Varki and Angata, 2006; Varki, 2009). Treatment of a microglial cell line and primary microglia with interferon-γ significantly enhances inflammatory phenotypes (e.g., up-regulation of iNOS, CXCL10 and TNFα) and up-regulation of Siglec-H. These Siglec-H-enriched microglia exhibit high phagocytosis activity that can be suppressed by down-regulation of Siglec-H. Importantly, Siglec-H binds to and triggers the engulfment of glioma cell lines (namely, GL261 and SMA560) but not normal astrocytes, splenocytes, or fibroblasts. It is possible that microglial Siglec-H may function to monitor the surface sialylation profiles present on tumor cells in the brain (Kopatz et al., 2013). These studies identified the functions of Siglec-H in the brain. However, whether enhancing Siglec-H activity is a potent approach to target brain tumor required further investigation.

### Siglec-H in ALS

Microglia share many markers with peripheral monocytes and macrophages. Transcriptome analysis reveals that Siglec-H is the third highest gene (out of 29 identified genes) that can distinguish microglia from monocytes and macrophages. The level of Siglec-H is also up-regulated in the spinal cord of an ALS mouse model (SOD1; Chiu et al., 2013). The function of Siglec-H in ALS remains unclear.

## C-Type Lectins

C-type lectins are a group of proteins that bind to glycan in a calcium-dependent manner. The proteins contain a C-type lectin domain (CTLD), which is also present in many proteins that do not recognize or bind to any glycan. C-type lectins and protein with CTLD are found in all organism and can be categorized to at least 17 groups based on their domain architecture, such as selectins, collectins, etc. The classification of C-type lectins and their roles in the peripheral immunity have been reviewed extensively elsewhere (Sancho and Reis e Sousa, 2012; Dambuza and Brown, 2015; Yan et al., 2015). In this review, we focus on a few C-type lectins (Table 6) that have been implicated in neurological disorder.

**Table 6:** Summary of the role of C-type lectins in neurological disease.

Neurological conditions	Type of C-type lectin	Disease-associated features	References
Stroke and ischemia	E-Selectin	Facilitates the adhesion of neutrophils to endothelial cells and promotes cell damage.	Zhang et al. (1996) and Jin et al. (2010)
	P-Selectin	Facilitates the adhesion of neutrophils to endothelial cells and promotes the activation of complement. Contributes to the breakdown of the BBB.	Atkinson et al. (2006) and Jin et al. (2010)
	Mannose-binding lectin	Up-regulated and contributes to cerebral infarction. Contributes to complement activation and promotes inflammation.	Cervera et al. (2010), Morrison et al. (2011), Orsini et al. (2012) and De Blasio et al. (2017)
Subarachnoid hemorrhage	P-Selectin	Recruits neutrophils and subsequently activates microglia through intravascular inflammation.	Atangana et al. (2017)
HIV infection	Mannose-binding lectin	Up-regulated in microglia, astrocytes, oligodendrocytes and neurons. Expression is associated with MCP-1, which may trigger inflammation.	Singh et al. (2011)

### Selectins

Selectins are single-chain transmembrane glycoproteins of the cell adhesion molecule family. They are highly homologous proteins, which contain a CTLD, an epidermal growth factor-like domain, a few consensus repeats composed of sushi domain, a transmembrane domain and an intracellular cytoplasmic tail. The CTLD of selectins recognizes sialyl Lewis X antigens (sialylated and fucosylated glycoprotein and glycolipid structures; Ley, 2003; Angiari and Constantin, 2013). Three types of selectins (i.e., E-, L- and P-Selectins) have being identified so far. In the brain, selectins (particularly the E- and P-type) are located in endothelial cells and are responsible for controlling leukocyte infiltration and the BBB permeability during trauma or acute inflammation (Tang et al., 1996; Carvalho-Tavares et al., 2000; Bernardes-Silva et al., 2001). This is an important aspect because accumulation and adhesion of leukocytes to the endothelial cells are the critical steps that cause cell death at the ischemic regions (Kataoka et al., 2004; Langer and Chavakis, 2009).

In the animal model of focal cerebral ischemia and reperfusion injury, E-Selectins are induced and expressed on the endothelial cells (Wang et al., 1995; Zhang et al., 1996). Because E-Selectins bind sialyl Lewis X glycans enriched in neutrophils (Lasky, 1992) and the administration of sialyl Lewis X significantly decreases the recruitment of neutrophils to the ischemic region and decrease the infarct volume (Zhang et al., 1996), E-Selectins appear to be an important component of ischemia pathogenesis. Another intriguing observation is that the polysialylated E-Selectin Ligand-1 (ESL1, a ligand for E-Selectins) was found to be enriched in the Golgi compartments of microglia. Brain injuries translocate ESL1 to the cell surfaces and therefore trigger the binding of E-Selectins with microglia (Werneburg et al., 2016). Binding of E-Selectins to myeloid cells (i.e., neutrophils) has been demonstrated to regulate cell homing to sites of inflammation (Mondal et al., 2016), while the effect of binding of E-Selectins to microglia remains to be determined.

**Figure 5:**
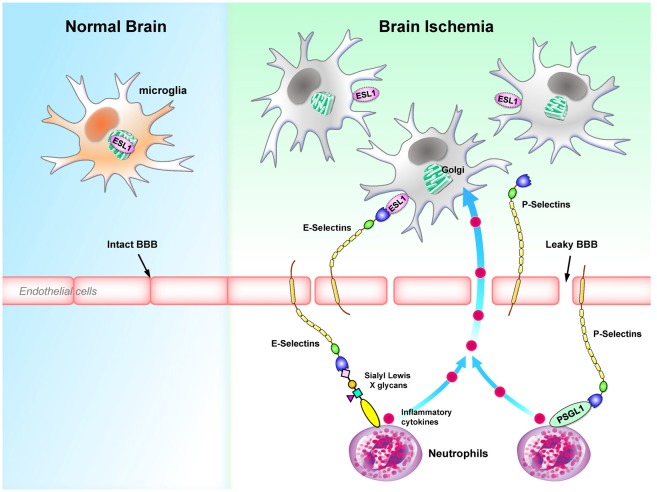
Selectins regulates microglial activation and homing to sites of inflammation during brain ischemia. E-selectins and P-selectins that bind to Sialyl Lewis X glycans are up-regulated in endothelial cells during brain ischemia. Binding of selectins serves as a homing mechanism to retain Sialyl Lewis X glycans-containing cells close to endothelial cells. In particular, ESL1 located in the Golgi compartments of microglia is translocated to the plasma membrane and binds to E-selectins on the surface of endothelial cells (Werneburg et al., 2016). In contrast, P-selectins cause a transient leakage of BBB (Jin et al., 2010). P-selectins bind to PSGL1 on neutrophils, which secrete inflammatory cytokines and further activate microglia in the CNS (Schneider et al., 2015; Atangana et al., 2017). BBB, blood-brain barrier; ESL1, E-selectin ligand-1; PSGL1, P-selectin glycoprotein ligand-1.

The expression of P-Selectin in endothelial cells is also up-regulated in a complement C3-dependent manner in an ischemia mouse model (Atkinson et al., 2006). Both P-Selectin deficiency and P-Selectin inhibition protect mice from the cerebral injury following ischemia (Zhou et al., 2000; Atkinson et al., 2006). Up-regulation of P-Selectin also contributes to the transient breakdown of BBB following ischemia (Jin et al., 2010). It is interesting to note that the level of P-Selectin is significantly up-regulated in the experimental subarachnoid hemorrhage (eSAH) model as well. The presence of P-Selectins on endothelial cells recruit neutrophils and microglia to the site of injury. It is likely that these microglia are activated by the inflammatory cytokines released by the neutrophils promoted by intravascular inflammation (Schneider et al., 2015; Atangana et al., 2017). In addition, induction of eSAH in mice lacking the P-Selectin glycoprotein ligand-1 (PSGL1; a P-Selectin ligand) results in significant reductions in the endothelial-neutrophil interactions, the number of activated microglia and the extent of neuronal death (Atangana et al., 2017). Collectively, these studies suggest that P-Selectin is likely to play a detrimental role in neurological traumas by facilitating the microglia-mediated neuroinflammation. Figure 5 summarizes the roles of E-selectins and P-selectins in brain ischemia.

### Mannose-Binding Lectins (MBLs)

MBLs, also called mannan-binding lectins, are from the collectin class of the C-type lectin family and are important components of the innate immune system. These proteins contain a cysteine-rich N-terminal region, a collagenous domain, a short α-helical coiled-coil domain and a CRD at the C-terminal. It is important to note that CRDs of MBL recognize glycans on the surface of pathogens as well as cryptic self-antigens that are modified in disease conditions or injuries (Ip et al., 2009). They exist in oligomeric forms (i.e., from dimer to hexamer) and recognizes mannose, fucose and N-acetyl-glucosamine (GlcNAc), but not sialic acid and galactose (Drickamer, 1992; Weis et al., 1992; Yokota et al., 1995). MBLs participate in a range of biological functions including the recognition of pathogen invasion, activation of complement system, phagocytosis and mediation of inflammatory signaling (Ip et al., 2009; Auriti et al., 2017).

Activation of complement system is one of the main causes for brain damage during ischemia and stroke. MBL has been implicated to participate in the activation of complement system via the lectin pathway (Morrison et al., 2011). MBL-KO mice are protected from transient and permanent ischemic injuries with the reduction of infarct volume and sensorimotor impairments. Blocking MBL using an anti-MBL-A antibody also produce similar results (Orsini et al., 2012; Longhi et al., 2014). Consistently, ischemic stroke patients, who have MBL-low genotypes and lower MBL in their serum, are associated with a favorable stroke outcome and decreased levels of proinflammatory cytokines (e.g., TNFα and IL6). These patients with lower MBL levels (<100 ng/ml) are likely to recover from movement disabilities in less than 3 months (Cervera et al., 2010; Osthoff et al., 2011). Collectively, effective inhibition of MBL may reduce the damage caused by ischemic stroke.

In patients infected by HIV, viruses can enter the CNS and continue to replicate within the brain. Infection of HIV in the brain therefore causes a chronic inflammatory response and a spectrum of neurological dysfunction (Saylor et al., 2016). By studying post-mortem brain tissues, it was shown that the expression of MBL is up-regulated in the HIV- infected brains. Interestingly, the expression of MBL is detected in all brain cells, including microglia, astrocytes, oligodendrocytes and neurons. In particular, axons in the HIV-infected brain show high levels of MBL, while the level of MBL is associated with the level of monocytes chemoattractant protein-1 (MCP-1 or CCL2; Singh et al., 2011). MCP-1 is the key chemokine that triggers the migration of microglia or macrophages (Deshmane et al., 2009; Hinojosa et al., 2011). Hence, the expression of MBL is suggested to be associated with the activation of complement and neuroinflammation, which subsequently cause neuronal damage in the HIV-infected brain.

## Conclusion and Future Perspectives

Accumulated evidence suggest that dysregulation of lectins is a critical pathogenetic factor in neurological diseases. The altered expressions of lectins on microglia affect the phenotypes of microglia (Yip et al., 2017). Importantly, the aberrant expression of lectins on the surrounding cells can also trigger the activation of microglia (Atangana et al., 2017). These may independently or synergistically lead to the chronic pathogenic inflammatory response that causes neuronal dysfunctions or death. Likewise, these activated microglia may also secrete inflammatory cytokines and complement that will influence the activity of astrocytes to produce neurotoxic factors (Liddelow et al., 2017).

One major area that still requires clarification is the specific type of cells that express these lectins. Many lectins are not expressed in the healthy CNS, nevertheless are induced to express or up-regulated in disease condition. It is crucial to identify the specific types of cells that show aberrant expression of lectins and their functional effect on the cells themselves or the surrounding cells. For instance, galectin-3 is expressed virtually on activated microglia in various neurological disorders *in vivo*, while it is expressed in various cell lines derived from brain glial cells *in vitro* (Pasquini et al., 2011). These studies raise the questions of why cell lines express galectin-3 even under normal conditions. There is a possibility that galectin-3 is involved in the immortalization and proliferation of cell lines, as it does in tumor cells (i.e., pancreatic cancer cells, thyroid carcinoma cells; Song et al., 2012; Cardoso et al., 2016). At the same time, it is also of interest to investigate whether other glial cells (astrocytes and oligodendrocytes) in abnormal conditions also express galectin-3 *in vivo*. Meanwhile, Galectin-4 is expressed in non-myelinated region of axons and Siglec-4 is expressed specifically on oligodendrocytes to regulate myelination (Figure 1). Contrary to Galectins that can be expressed in various types of cells, all CD33-related Siglecs are expressed specifically on myeloid- origin cells, namely, microglia in the CNS. Selectins are mainly on endothelial cells and their expressions directly affects the integrity of BBB and the activity of nearby microglia. MBL has been reported to increase in stroke and ischemia, while the sources of MBL are elusive. It is unclear whether MBLs are produced by brain cells or migrate from the peripheral nervous system due to the leakage of the BBB. Interestingly, in HIV-infected brain, all brain cells show positive signals for MBL, indicating brain cells are capable of producing MBL upon stimulation. Further understandings on how lectins affect microglia and its interaction with other brain cells certainly would broaden our view on neurodegenerative diseases and raise new questions as well. Are these lectins druggable targets for neurological disease? Would pharmacological inhibition or activation of these lectins be effective in treating neurological diseases? Notably, these inhibitors/activators also need to have the capacity to cross the BBB to treat brain disorders.

The concept of lectin-based therapeutics deploys the specific targets of lectins or the delivery of drugs that interacts with lectins to the target site. For example, Galectin-3 has been implicated in numerous neurological disorders and diseases in the peripheral nervous system. Pharmacological suppression of galectin-3 appears to be a promising approach. Specifically, TD139 that binds to galectin-3 and inhibit its functions have passed the clinical phase Ib/IIa trials for treating idiopathic pulmonary fibrosis (http://ClinicalTrials.gov identifier, NCT02257177). Although this development is encouraging, the ability of TD139 to cross the BBB is poor and therefore limits its application to neurological diseases with abnormal up-regulation of Gal3. Further modification of TD139 to increase its permeability across the BBB may pave the way for the development of therapeutic intervention for brain diseases.

On the other hand, lectin-based therapeutics may enhance the exposure of drugs to the targeted cells/sites, and reduce the dosage and side effects. Moreover, internalization of drug also increases the uptake of therapeutic agents with low cellular permeability. The decoration of drug enveloped with surface sialic acid ligands can directly activate Siglecs downstream signaling or deploy their internalization properties. For example, nanoparticles coated with di(α2–8) N-acetylneuraminic acid effectively target Siglec-7 and Siglec-9 to exert anti-inflammatory effects of macrophages in a human *ex vivo* lung perfusion model (Spence et al., 2015). Furthermore, liposomal nanoparticles that are coated with high-affinity CD22 ligands result in substantial uptake of the nanoparticles by B cell lymphomas that express the endocytic receptor CD22 (Chen et al., 2012).

Collectively, emerging studies have shown that modulating the lectins activity via direct interference in the lectin itself or the receptor-ligand interaction in CNS is a promising area for further exploration. Currently, treatment of neurological disorders using a lectin-based approach has not been envisaged, likely owing to an incomplete understanding of the roles of lectins in the CNS and the limited ability of drugs to cross the BBB. A greater understanding of microglial lectins may facilitate the development of novel therapeutic interventions for patients with neurological disorders.

## Author Contributions

JJS designed and wrote the manuscript. YC designed and edited the manuscript.

## Conflict of Interest Statement

The authors declare that the research was conducted in the absence of any commercial or financial relationships that could be construed as a potential conflict of interest.
